# Case report: A rare case of retroperitoneal mixed type unicentric Castleman disease

**DOI:** 10.3389/fonc.2026.1796146

**Published:** 2026-05-01

**Authors:** Jiajie Zhang, Jian Zhang, Jinhao Liu, Panying Zhang, Shoubin Li

**Affiliations:** 1 Hebei North University, Zhangjiakou, Hebei, China; 2 Department of Urology, Hebei General Hospital, Shijiazhuang, Hebei, China

**Keywords:** diagnosis, laparoscopic surgery, mixed type unicentric Castleman disease, prognosis, retroperitoneal mass, therapy, treatment

## Abstract

**Objective:**

To enhance the understanding, diagnosis, and management of retroperitoneal mixed type unicentric Castleman disease.

**Methods:**

This report describes a case of retroperitoneal mixed type unicentric Castleman disease managed at our institution. The clinical presentation, histopathological characteristics, and therapeutic approach are detailed. A discussion regarding the current status of diagnosis, management, and prognosis is provided, supplemented by a review of the relevant literature.

**Results:**

A 52-year-old female was admitted with a chief complaint of “frequent urination for over 6 months.” A retroperitoneal mass was identified. The patient underwent laparoscopic excision of the retroperitoneal mass. Postoperative pathological examination confirmed the diagnosis of mixed type unicentric Castleman disease. The patient’s postoperative recovery was uneventful, and she remains under close follow-up surveillance.

**Conclusion:**

Retroperitoneal mixed type unicentric Castleman disease is clinically uncommon and possesses distinctive histopathological features. Awareness of this entity needs to be heightened.

## 1 Introduction

Castleman disease, also known as vascular follicular lymphoid hyperplasia or giant lymphadenopathy, is a rare benign lymphoproliferative disorder (1). The incidence and prevalence of the disease are unclear; but one study showed the incidence of all forms of CD to be 21 in one million, with estimated cases in the United States ranging from 30–000 to 100–000 (2). UCD mainly occurs in the third and fourth decades of life, with an average age of 35 years, and a slight predilection to females. In approximately 11–12% of cases, Castleman’s disease (CD) manifests in the retroperitoneum (2). Although unicentric Castleman disease (UCD) is predominantly a benign lesion, its clinical manifestations are diverse and nonspecific, rendering it highly susceptible to confusion with other lymphoproliferative disorders and thereby leading to misdiagnosis or missed diagnosis. Therefore, it is of great significance to conduct an in-depth exploration of its research progress and to emphasize the clinical value of rare case reports. This report presents a case of retroperitoneal mixed type unicentric Castleman disease, discussing its clinicopathological features and diagnostic-therapeutic experience.

## 2 Case presentation

A 52-year-old female was admitted due to a 6-month history of urinary frequency. The symptom onset was without apparent cause. She reported no accompanying urinary urgency, dysuria, hematuria, fever, nausea, vomiting, or weight loss. Laboratory investigations upon admission revealed no significant abnormalities.

**Figure 1:**
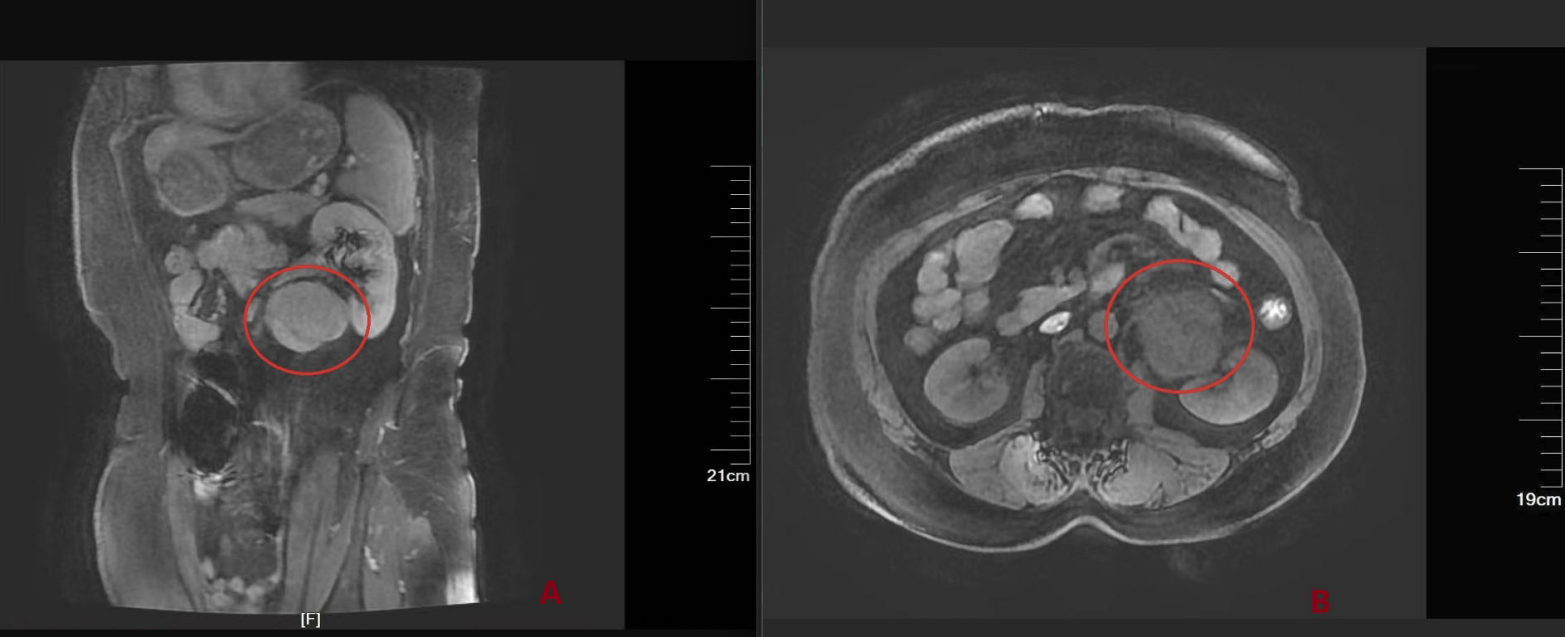
MRI: A circular mass can be seen in front of the left kidney in the retroperitoneal area, no 182 invasion is observed. **(A)** Sagittal plane. **(B)** Transverse plane. Before the surgery, we performed ultrasound−guided percutaneous needle biopsy on the patient. As shown in **Figure 2**. The ultrasound−guided percutaneous needle biopsy result showed CD21(+), CD79a(+), CD20(+), CD34(+), CD38(+), and CD138(+), suggested Unicentric Castleman Disease.

Computed Tomography (CT) and magnetic resonance imaging (MRI) demonstrated a round mass anterior to the left kidney in the retroperitoneal space. As shown in **Figure 1**. It showed hypointense signal on T1WI and heterogeneous hyperintense signal on T2WI, with high signal intensity on DWI and corresponding low signal intensity on the ADC map. The lesion measured approximately 47 × 46 × 51 mm and demonstrated marked heterogeneous enhancement in the early phase. Prominent heterogeneous enhancement was observed in the early contrast phase. The initial diagnosis was a retroperitoneal mass.

**Figure 2:**
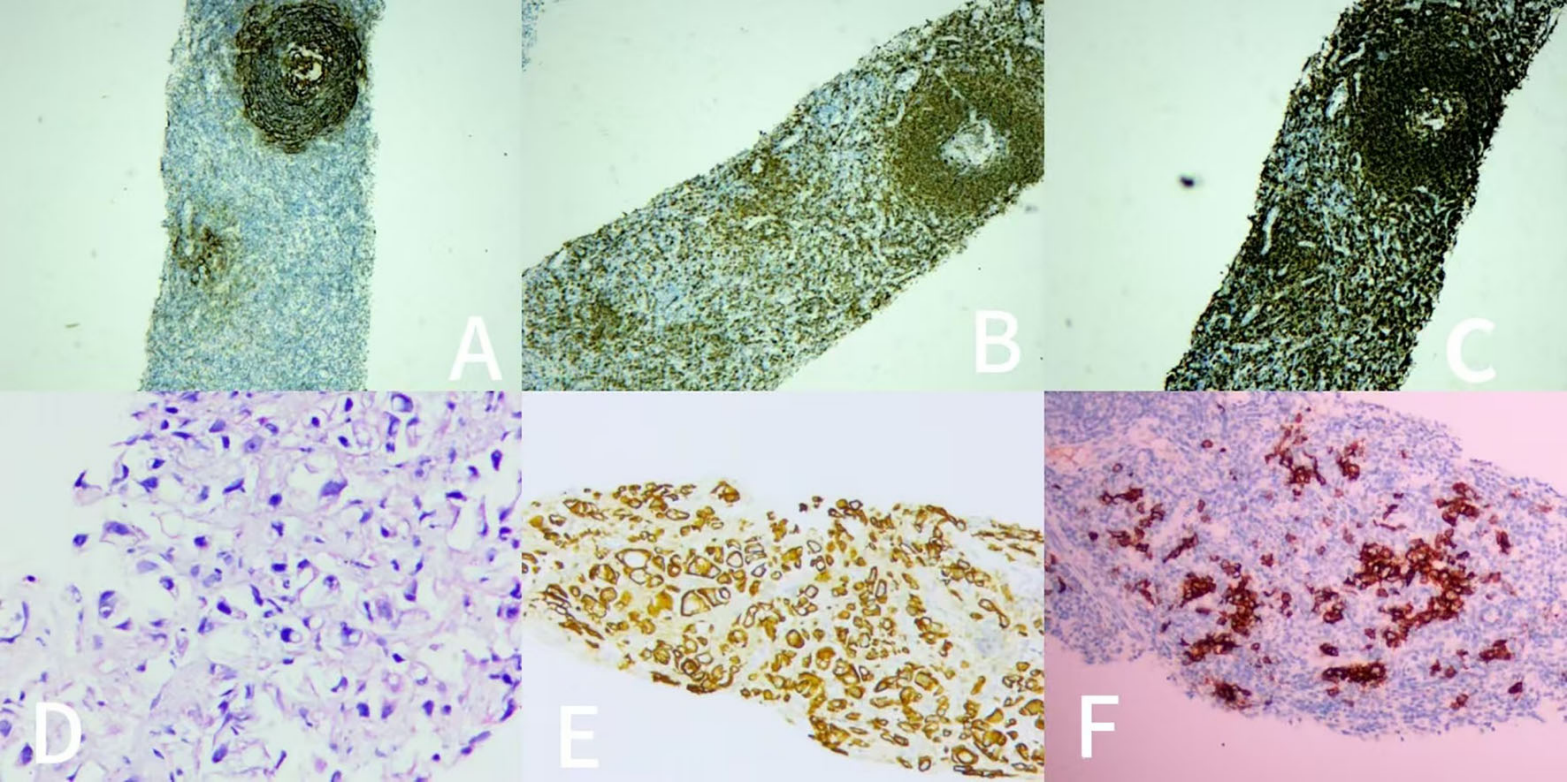
Ultrasound−guided percutaneous needle biopsy:CD21(+); CD79a(+); CD20(+); CD34(+); CD38(+); CD138(+). Representative histopathologic and immunohistochemical findings in a patient with unicentric Castleman disease (mixed type). **(A)** The immunohistochemical stain, ×100: CD21 (follicular dendritic cells). **(B)** The immunohistochemical stain, ×100:CD79a(B cell proliferation). **(C)** The immunohistochemical stain, ×100:CD20(B cell proliferation). **(D)** The immunohistochemical stain, ×100:CD38(clonal expansion of plasma cells). **(E)** The immunohistochemical stain, ×100:CD34 (proliferated venules). **(F)** The immunohistochemical stain, ×100:CD138 (clonal expansion of plasma cells).

**Figure 3:**
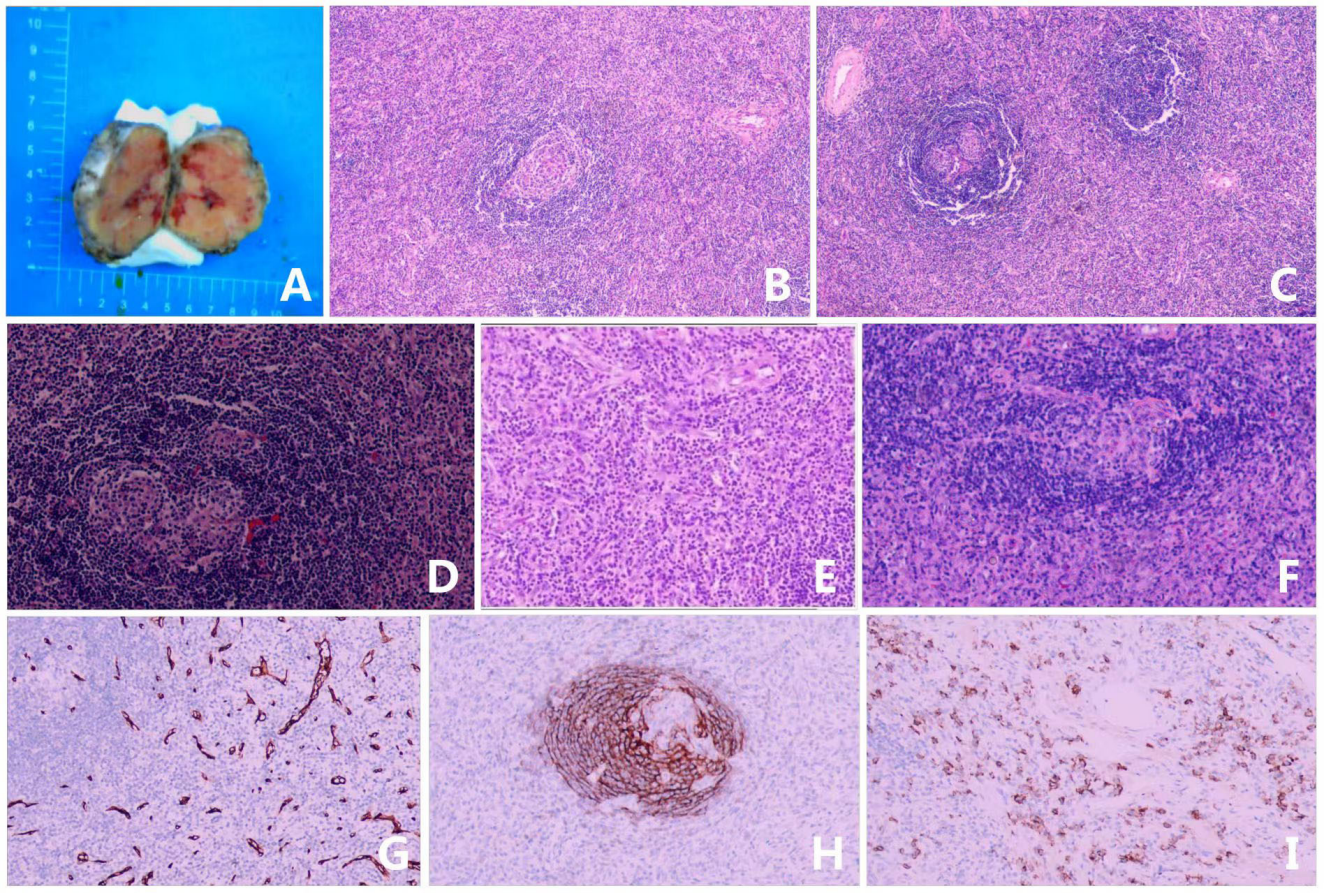
Representative histopathologic and immunohistochemical findings in a patient with unicentric Castleman disease (mixed type). **(A)** Gross, microscopic, and immunohistochemical features of the tumor. **(B)** H&E stain, ×100: lymph node with lymphoid cells in an “onion skin” pattern with a hyaline center. **(C)** H&E stain, ×100: Enhanced expression in germinal center B cells, with well-defined follicular boundaries. **(D–F)** H&E stain, ×200: The lymph nodes exhibited follicular hyperplasia, and the interfollicular areas were characterized by a rich presence of plasma cells accompanied by vascular proliferation. Furthermore, there was evidence of degeneration in the lymphoid follicles. A mixture of plasma cell (PC) and hyaline vascular (HV) features was observed, confirming a mixed type. **(G)** The immunohistochemical stain, ×100: CD34 (proliferated venules). **(H)** The immunohistochemical stain, ×100: CD21 (follicular dendritic cells). **(I)** The immunohistochemical stain, ×100: CD138 (clonal expansion of plasma cells). The patient’s postoperative recovery was uneventful. At a 3-month follow-up outpatient visit at our institution, she remained asymptomatic with no evidence of disease recurrence.

**Figure 4:**
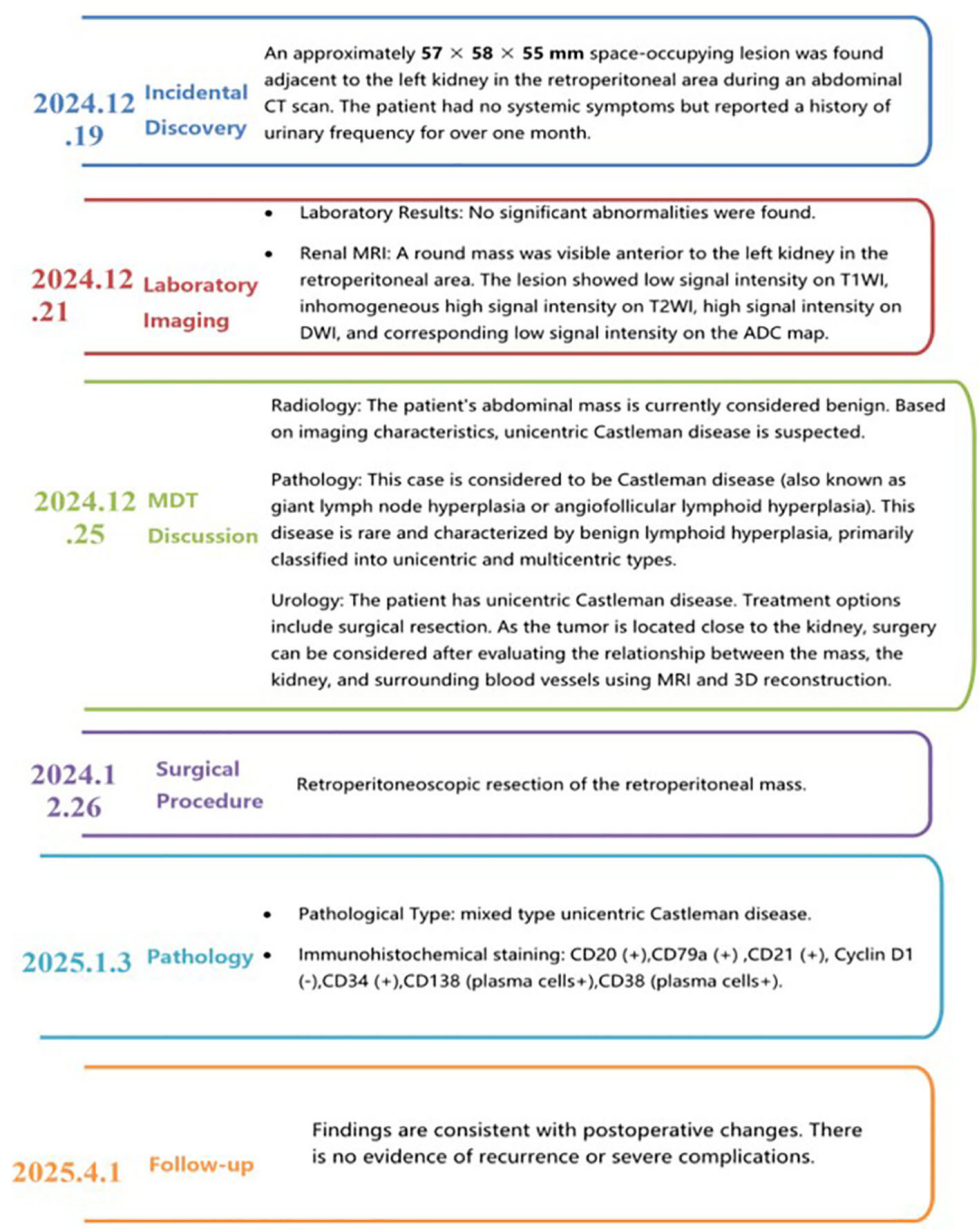
Treatment timeline of this case.

Following an evaluation that revealed no surgical contraindications, the patient underwent a laparoscopic resection of the retroperitoneal mass. The excised specimen measured approximately 5.5 x 5.3 x 3.8 cm, with a soft, tan-reddish cut surface. Histopathological analysis revealed reactive hyperplasia of lymphoid tissue, characterized by widened follicular mantles, regressed germinal centers, and a prominent interfollicular infiltrate of plasma cells. As shown in **Figure 3**. Immunohistochemical staining yielded the following results: CD20 (+), CD79a (+), CD21 (+), Cyclin D1 (-), CD34 (+), CD138 (plasma cells+), CD38 (plasma cells+). The treatment timeline of this case is shown in **Figure 4**.

## 3 Discussion and conclusion

Castleman disease (CD) comprises a spectrum of rare and heterogeneous disorders characterized by distinct lymph node histopathological abnormalities. CD may involve a single lymph node station, classified as unicentric Castleman disease (UCD), or present with systemic lymphadenopathy and inflammatory symptoms, designated as multicentric Castleman disease (MCD) (3). Histological variants include the hyaline vascular (HV) type, plasma cell (PC) type, and mixed type (4). The etiology of CD remains poorly elucidated. UCD is often challenging to diagnose preoperatively. When located in the retroperitoneum, it is frequently misdiagnosed as a teratoma, dermoid cyst, lymphoma, retroperitoneal sarcoma, schwannoma, pheochromocytoma, or paraganglioma (5). Definitive diagnosis relies on postoperative pathological examination or needle biopsy. UCD patients appear to have a higher risk of developing follicular dendritic cell sarcomas and Hodgkin or nonHodgkin lymphoma (6, 7).The possibility of UCD should be considered when a solid mass is detected in the abdomen or retroperitoneum.

UCD typically manifests as isolated lymphadenopathy, often with minimal or localized symptoms. In its early stages, it is frequently asymptomatic, making preoperative diagnosis and differential diagnosis challenging and leading to a high rate of misdiagnosis. In contrast, MCD presents with diverse systemic manifestations, including fever, weight loss, anasarca (i.e., severe generalized edema), and generalized lymphadenopathy frequently accompanied by splenomegaly (which can be massive) and, in some cases, hepatomegaly. The involved lymph nodes are usually widespread but relatively small in size, with massive nodal enlargement being uncommon. Anemia and hypoalbuminemia are highly prevalent, and other cytopenias may also be observed (8).

In UCD, the hyaline vascular (HV) histopathologic subtype is most frequently observed. Patients with this subtype are typically devoid of systemic symptoms or cytokine excess. Lymph node architecture demonstrates follicular hyperplasia with regressed germinal centers, increased vascularity with hyalinization, prominent and/or dysplastic follicular dendritic cells (FDCs), and expanded mantle zones with an “onion-skin” appearance. Additional characteristic findings include radially penetrating vessels creating a “lollipop” appearance, multiple germinal centers within a single mantle zone (described as “twinning” or “floral”), tight aggregates of plasmacytoid dendritic cells, and overall architectural disarray with sinus obliteration. Plasma cell (PC) histopathologic subtype accounts for only approximately 10–20% of UCD cases. It is characterized by variably sized germinal centers and interfollicular plasmacytosis, occasionally associated with systemic inflammatory symptoms (3). Of note, Morita-Hoshi, et al. (9) reviewed 45 cases of systemic amyloidosis related to CD and showed that: The plasma cell type was the most common histology in patients either with unicentric or multicentric disease complicated with amyloidosis. Almost all cases (95%) in which the type of amyloidosis was confirmed had the AA type (10). MCD is an infrequent and life-threatening disorder (11). Its pathological features may resemble those observed in UCD and lack specificity, exhibiting substantial overlap with entities including chronic infections, autoimmune diseases, and hematologic malignancies, thus complicating the diagnostic process.

The optimal management strategy for UCD remains to be fully standardized; however, surgical resection is established as the gold standard therapeutic intervention. Following complete surgical excision, the cure rate is notably high, with long-term survival comparable to that of the healthy population (12). In contrast, MCD carries a less favorable prognosis. For multicentric disease, and specifically non-severe idiopathic MCD (iMCD), siltuximab is the first-line pharmacologic therapy. For selected patients with limited symptomatology, a short-course regimen of rituximab may serve as an alternative option. The management of severe iMCD is particularly challenging and often necessitates early initiation of combination chemotherapy to avert potentially fatal outcomes (13).

The heterogeneous clinical presentations of UCD often pose significant challenges in diagnosis and management. A deeper understanding of this disease and its characteristics will help surgeons avoid unnecessarily extensive resection of this non-malignant condition when encountering abdominal or retroperitoneal masses (14).

## Data Availability

The original contributions presented in the study are included in the article/supplementary material. Further inquiries can be directed to the corresponding author.
